# The global prevalence of familial multiple sclerosis: an updated systematic review and meta-analysis

**DOI:** 10.1186/s12883-021-02267-9

**Published:** 2021-06-28

**Authors:** Naeim Ehtesham, Maryam Zare Rafie, Meysam Mosallaei

**Affiliations:** 1 Student Research Committee, University of Social Welfare and Rehabilitation Sciences, Koodakyar Alley, Daneshjoo Blvd., Evin St, Tehran, Iran; 2 Department of Genetics and Molecular Biology, School of Medicine, Isfahan University of Medical Sciences, Isfahan, Iran; 3 School of Medicine, Zanjan University of Medical Sciences, Zanjan, Iran

**Keywords:** Familial multiple sclerosis, Pediatric-onset multiple sclerosis, Systematic review, Meta-analysis

## Abstract

**Background:**

Considering that many recent studies have reported the prevalence of familial multiple sclerosis (FMS), we performed an updated meta-analysis of the worldwide prevalence of FMS by the addition of recent publications.

**Methods:**

A search in PubMed, Scopus, the ISI Web of Science, and Google Scholar was undertaken up to 20 December 2020. The inclusion criteria were based on the CoCoPop approach (condition, context, and population). Meta-analysis of the qualified studies was conducted by comprehensive meta-analysis ver. 2 software.

**Results:**

The pooled prevalence of MS in relatives of 16,179 FMS cases was estimated to be 11.8% (95% CI: 10.7–13) based on a random-effects model. The pooled mean age of disease onset in adult probands was calculated to be 28.7 years (95% CI: 27.2 ± 30.2). Regarding 13 studies that reported the data of FMS in pediatrics (*n* = 877) and adults (*n* = 6636), the FMS prevalence in pediatrics and adults was 15.5% (95% CI: 13.8–17.4) and 10.8% (95% CI: 8.1–14.2), respectively. The prevalence of FMS in affected males (*n* = 5243) and females (*n* = 11,503) was calculated to be 13.7% (95% CI: 10.1–18.2) and 15.4% (95% CI: 10.3–22.4), respectively. The odds ratio of male/female in FMS cases was not statistically significant (OR = 0.9; 95% CI: 0.6–1.2, *P* = 0.55). Subgroup analysis demonstrated a significant difference in the prevalence of FMS between the geographical areas (*P* = 0.007). The meta-regression model indicated that the prevalence of FMS is lower with higher latitude and higher MS prevalence (*P* < 0.001). In contrast, meta-regression based on prevalence day was not statistically significant (*P* = 0.29).

**Conclusions:**

The prevalence of FMS is higher in the pediatric group than that of adults, distinct between geographical areas, and diminishes with the increment of MS prevalence and latitude. Also, the symptoms initiate relatively at younger ages in the FMS cases. Interestingly, our analysis unveiled that FMS is not more prevalent in men than women and the risk of MS development in relatives is not higher when the affected proband is male.

**Supplementary Information:**

The online version contains supplementary material available at 10.1186/s12883-021-02267-9.

## Background

Multiple sclerosis (MS), chronic inflammatory demyelinating disorder of the central nervous system, is the most common cause of non-traumatic neurological disability in a range of age groups especially young adults and afflicts more than 2.5 million individuals in the world [1]. Both genetic variations and environmental factors partake synergistically in the development of MS. The effect size of genetic risk factors ranges from small to modest. For instance, HLA-DRB1*15:01, as the major genetic determinant, has an odds ratio (OR) ~ 3.5, which increases to 8 in homozygous carriers [2, 3] The identified environmental risk triggers for MS development subsumes distance from the equator (latitude), vitamin D deficiency, lack of sunlight, smoking, obesity, and most importantly infection by Epstein-Barr virus, which is the largest environmental risk (OR ~ 3.6) [4]. The heterogeneous distribution of MS in distinct populations has been attributed to the interplay between different genetic backgrounds and environmental exposures [5].

The earliest report of the existence of MS in more than one family member (familial MS or FMS) dates back to 1938 [6]. In the years since then, many studies have revealed the prevalence of FMS in many populations. All of them considered FMS as the occurrence of the same disease in at least one any-degree relative of patients. However, two nationwide register-based studies in Denmark did not consider the presence of MS in distant relatives comprising 2nd or 3rd-degree relatives as FMS cases [7, 8].

In this study, we aimed to perform an updated systematic review and meta-analysis about the worldwide prevalence of FMS by the addition of new studies. In contrast to the previous study [9], we conducted a separate meta-analysis on the prevalence of FMS in pediatric-onset MS (POMS) and adult-onset MS (AOMS) as well as men and women. Also, subgroup analysis based on geographical area, and meta-regression based on latitude, prevalence date, and MS prevalence was conducted. In addition, we accomplished a meta-analysis of sex ratio and mean age of onset among FMS cases.

## Methods

Preferred Reporting Items for Systematic Reviews and Meta-Analyses (PRISMA) guidelines [10] were recruited to perform the present systematic review focusing on the prevalence of FMS in the world. Each process of research was done independently by two investigators and disagreements were resolved by discussion with the third author.

### Search strategy

We accomplished a comprehensive search in PubMed, Scopus, and the ISI Web of Science up to 20 December 2020. Boolean operators (AND & OR) were utilized to search by a combination of these keywords: “multiple sclerosis”, “familial”, “epidemiology”, “prevalence”, “incidence”, “recurrence” and “frequency”. The details of the search strategy are documented in Additional file 1. Finally, Google Scholar was searched to find further works. No language or date restriction was applied to the literature search. We manually checked the reference lists of obtained articles to not miss any additional documents.

### Eligibility criteria

For defining the criteria for inclusion and exclusion of studies, we employed the CoCoPop approach (condition, context, and population) which is used for systematic reviews of prevalence studies [11]. According to this approach, the original studies with available full-text that have investigated the prevalence of MS in full biological relatives of patients with definite MS (not probable, possible, or suspected), and have been conducted in a specific region, time, and target population were enrolled. The reason behind the criteria for definite MS is that some neurological disorders mimic MS. The studies in the same region but with different time periods and sample frames were also included. Studies with duplicate data were excluded.

### Data extraction

By using a pre-prepared sheet, these data were collected from the eligible studies: first author’s last name, publication year, prevalence day or period (the time point or period that the FMS prevalence was determined), setting and case ascertainment, the place of the research, diagnostic criteria of probands, the method for the ascertainment of MS in relatives, the number of FMS cases and total patients, mean age of disease onset in probands, the prevalence of MS, the number of POMS in FMS cases, geographical area, and sex ratio of probands. For providing insight into the difference between the prevalence of FMS in adults and pediatrics, the studies that reported FMS prevalence in AOMS and POMS separately were considered as two different data sets.

### Quality assessment

For assessing the methodological quality of included studies, Joanna Briggs Institute’s critical appraisal tool was exploited which comprises 9 questions [12]. If the answer to a question was “Yes”, 1 score was considered. Points 0–5, 6–7, and 8–9 were regarded as low, moderate, and high quality, respectively. The minimum score for enrolment of the studies was 5. This was because, in some of the studies with a 5 score, one or more quality parameters were ambiguous. In other words, these studies had borderline quality and could be regarded as studies with moderate quality.

### Statistical analysis

For choosing between random-effects and fixed-effects models, heterogeneity of studies was evaluated by Cochran’s Q and I^2^ tests. For verification of the stability of data, a sensitivity analysis was performed. In addition to a meta-analysis of the prevalence of FMS in all studies, a separate analysis was implemented on studies that reported, separately, the prevalence of FMS in AOMS and POMS cases and males and females. By using the number of FMS and total MS in male and female groups, we calculated the odds ratio (OR) and 95% CI of prevalence to estimate the effect of gender. To find the underlying cause of heterogeneity, subgroup analysis was performed based on geographical area and meta-regression was carried out in terms of latitude, MS prevalence, and prevalence day. We assessed the publication bias by using Begg and Egger’s tests. Comprehensive meta-analysis ver. 2 software was utilized for analysis and statistical significance was set at a *p*-value< 0.05.

## Results

### Literature search and characteristics

**Fig. 1:**
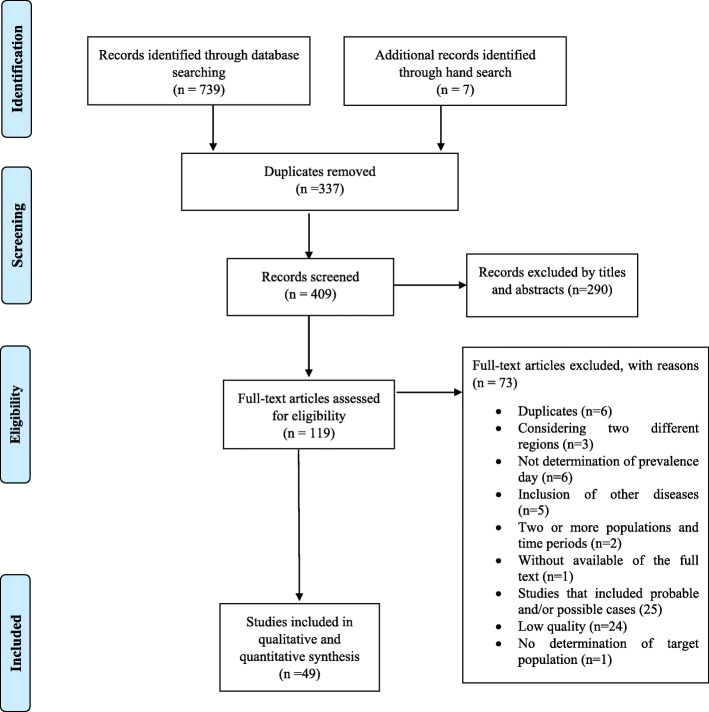
Flowchart of study selection through the systematic search

Collectively, database and manual search led to the finding of 739 and 7 records, respectively. Obtaining only 7 additional works by manual search indicates that our search strategy was robust and did not affect the integrity of our review. After removal of duplicates, initial screening was performed based on titles and abstracts which left 119 articles for assessment of the full-text. Of these, 73 articles were excluded for these reasons (Additional file 2): six had duplicate data, three considered more than one specific region, six did not determine the prevalence day, five reported the data in a combination of Neuromyelitis Optica (NMO), acquired demyelinating syndromes (ADS) and MS cases, two were performed in two or more populations and time periods, one without available of the full-text, 25 with the inclusion of probable and/or possible cases, 24 low-quality studies and one with no determination of the target population. Finally, 49 studies from 46 articles with a sample size of 16,179 FMS cases were included in our analysis (Fig. 1). The characteristics of these studies are represented in Table 1. The eligible articles have been published from 1984 to 2020 and regardless of six studies, the rest of included studies had a cross-sectional design.

**Table 1:** The characteristics of included studies

First author, Published Year^a^	Prevalence day or period	Setting/ case ascertainment	Place	Diagnostic criteria of probands	Tool for ascertainment of MS in relatives	Number of probands /Sample size	Mean age of MS onset in probands	FMS Prevalence (%)	MS Prevalence (Per 100,000)	Latitude^c^	POMS in probands (Below 18 or 16 yr.)	Geographical area	Sex ratio of probands (F/M)	Quality score
Salehi, 2020a [13]	1999–2018	Cross-sectional/ Iranian MS Society registry system	Tehran, Iran	McDonald^b^	Questionnaire	2506/ 18,945	28.49 ± 8.79	13.2	148	35°44′N	Nobody	Middle East	NR	9
Salehi, 2020b [13]	1999–2018	Cross-sectional/ Iranian MS Society registry system	Tehran, Iran	McDonald	Questionnaire	220/1391	NR	15.8	148	35°44′N	All < 18	Middle East	NR	9
Dorche, 2020 [14]	2004–2018	Cross-sectional/ Neurology clinic	Shiraz, Iran	McDonald	Medical records	48/871	M: 26.4 ± 9.7 F: 24.3 ± 10.5	5.5	63.4	29.59°N	NR	Middle East	NR	5
Ceccarelli, 2020 [15]	2015–2017	Cross-sectional/ Single-center Hospital	Abu Dhabi, UAE	McDonald	Questionnaire	24/98	28.9 ± 10.7	24.5	7	24°28′N	Nobody	Middle East	1.6	5
AlJumah, 2020 [16]	2015–2018	Cross-sectional/ National registry	Saudi Arabia	McDonald	Questionnaire	315/2465	26.81 ± 8.98	12.8	41	23.88° N	NR	Middle East	1.9	5
Steenhof, 2019a [7]	1960–2016	Cross-sectional/ Danish MS Registry	Denmark	Allison/Millar, Poser and/or McDonald	Medical records	1122/ 18,095	NR	6.2	282	56° 00 N	Nobody	Europe	1.9	6
Steenhof, 2019b [8]	1994–2014	Cross-sectional/ Danish MS Registry	Denmark	Poser and McDonald	Medical records	531/7402	NR	7.2	282	56° 00 N	Nobody	Europe	2.1	7
Mohebi, 2019 [17]	1989–2016	Cross-sectional/ Iranian MS Society registry system	Tehran, Iran	McDonald	Questionnaire	2260/ 18,061	28.03 ± 8.69	12.52	116	35°44′N	8.18 < 18	Middle East	2.80	8
Eskandarieh, 2019 [18]	1999–2017	Cross-sectional/ Iranian MS Society registry system	Tehran, Iran	Before 2001 = Poser After 2001 = McDonald	Questionnaire	288/1937	15.87 ± 2.28	14.9	148.06	35°44′N	All ≤18	Middle East	3.05	8
Abbasi, 2019 [19]	2017–2018	Cross-sectional/ Ardabil MS Registry	Ardabil, Iran	McDonald	Medical records	85/611	NR	14	59.37	38°15′N	NR	Middle East	2.7	6
Yamamoto, 2018 [20]	2002–2015	Cross-sectional/ academic institution	Cleveland, Ohio, USA	McDonald	Medical records	19/60	NR	32	288	41°30′N	All < 18	North America	NR	5
Viswanathan, 2018 [21]	2009–2017	Cross-sectional/ three major neurology departments (either clinics or wards)	Malaysia	McDonald	Medical records	6/123	NR	4.9	3	2°30′N	NR	Southeast Asia	NR	7
Omrani, 2018 [22]	2005–2015	Cross-sectional/ Iranian MS Society registry system	Tehran, Iran	McDonald	Questionnaire	50/300	NR	16.7	115.94	35°44′N	All < 18	Middle East	NR	7
Eskandarieh, 2018a [23]	1991–2017	Cross-sectional/ Iranian MS Society registry system	Tehran, Iran	Before 2001 = Poser After 2001 = McDonald	Questionnaire	2547/ 19,902	NR	12.8	148.06	35°44′N	NR	Middle East	NR	6
Eskandarieh, 2018b [24]	1999–2015	Cross-sectional/ Iranian MS Society registry system	Tehran, Iran	Poser (up to 2001) McDonald (after 2001)	Questionnaire	1793/ 13,968	NR	12.8	115.94	35°44′N	Nobody	Middle East	NR	8
Eskandarieh, 2018c [24]	1999–2015	Cross-sectional/ Iranian MS Society registry system	Tehran, Iran	Poser (up to 2001) McDonald (after 2001)	Questionnaire	242/1647	NR	14.7	115.94	35°44′N	All < 18	Middle East	NR	8
Eskandarieh, 2017a [25]	2014–2015	Cross-sectional / Iranian MS Society registry system	Tehran, Iran	McDonald	Questionnaire	207/1495	NR	13.8	101.39	35°44′N	NR	Middle East	NR	7
Eskandarieh, 2017b [26]	2013–2014	Cross-sectional/ Iranian MS Society registry system	Tehran, Iran	McDonald or Poser	Questionnaire	174/1234	NR	14.1	101.39	35°44′N	NR	Middle East	NR	6
Schiess, 2016 [27]	2010–2014	Cross-sectional/ Multi-center Hospital	Abu Dhabi, UAE	McDonald	Questionnaire	32/257	NR	12.4	64.44	24°28′N	NR	Middle East	NR	6
Rojas, 2016 [28]	2016	Cross-sectional/ 14 MS Centers	Argentina	McDonald, Poser	Questionnaire	97/1333	29.4 ± 5.1	7.3	25	34°00′S	NR	South America	1.5	8
Mazdeh, 2016 [29]	2013	Cross-sectional/ Single-center Hospital	Hamadan, Iran	McDonald	Questionnaire	103/1202	NR	8.57	61	34°52′N	NR	Middle East	3.2	5
Etemadifar, 2016a [30]	2003–2013	Cross-sectional/ Isfahan MS Society registry system	Isfahan, Iran	McDonald	Medical records	650/4315	NR	15	58.7	32°39′N	Nobody	Middle East	NR	6
Etemadifar, 2016b [30]	2003–2013	Cross-sectional/ Isfahan MS Society registry system	Isfahan, Iran	McDonald	Medical records	22/221	NR	9.9	58.7	32°39′N	All ≤16	Middle East	NR	6
Alifirova, 2016 [31]	1980–2014	Cross-sectional/ Tomsk MS registry system	Tomsk, Russia	McDonald	Medical records	15/320	26.05 ± 9.91	4.7	50	56°30′N	NR	Europe	NR	7
Abedini, 2016 [32]	2013–2014	Cross-sectional/ Mazandaran MS Society registry system	Mazandaran, Iran	McDonald	Questionnaire	33/152	NR	21.7	57	36°12′N	NR	Middle East	NR	6
Papais-Alvarenga, 2015 [33]	1990–2011	Cross-sectional/ Single-center Hospital	Río de Janeiro State, Brazil	McDonald	McDonald	40/653	28.5 ± 10.57	6.12	15	22° 55′ S	6 (15%) < 18	South America	4	9
Toghianifar, 2014 [34]	2011	Cross sectional/ Isfahan MS Society registry system	Isfahan, Iran	McDonald	Questionnaire	430/3911	29.7 ± 9.6	11	35	32°39′N	59 (13.7%) ≤19	Middle East	2.61	8
Reinhardt, 2014 [35]	2009–2011	Cohort/ German MS Society registry system	Germany	McDonald	Questionnaire	17/122	NR	13.9	210	51.51°N	All ≤15	Europe	NR	6
Hader, 2014 [36]	1977–2012	Cohort/ Local MS registry	Saskatoon, Saskatchewan, Canada	Allison and Millar,Schumacher	Questionnaire	49/150	M: 27.6 ± 8.4 F: 26.8 ± 8.5	32.7	110	52°10′N	NR	North America	2.8	7
Alroughani, 2014 [37]	2009–2012	Cross-sectional/ two MS clinics	Kuwait	McDonald	Medical records	98/736	NR	13.32	83	29° 30′ N	NR	Middle East	NR	7
Tipirneni, 2013 [38]	2006	Cross-sectional/ 15 MS treatment centers	New York, USA	McDonald	Questionnaire	196/758	36.2 ± 9.6	25.9	135	42°53′N	NR	North America	3.5	8
Saman-Nezhad, 2013 [39]	2012	Cross-sectional/ Kermanshah MS Society registry system	Kermanshah, Iran	McDonald	Medical records	14/448	NR	3.1	43.3	34°23′N	NR	Middle East	NR	6
Rezaali, 2013 [40]	2011	Cross-sectional/ Qom MS Society registry system	Qom, Iran	Poser and McDonald	Medical records and questionnaire	64/572	NR	11.2	50.4	34°40′N	NR	Middle East	NR	7
Hashemilar, 2011 [41]	2005–2009	Cross-sectional/ CDTMS registry	East Azerbaijan, Iran	McDonald	Medical records	71/1000	NR	7.1	27.7	37°20′N	NR	Middle East	1.6	6
Banwell, 2011 [42]	2004–2010	Cohort/ 16 pediatric health-care facilities and seven regional health-care facilities	Canada	McDonald	Questionnaire	10/63	NR	16	281	60° 00 N	All < 16	North America	NR	6
Ashtari, 2011 [43]	2007	Cross-sectional/ MS clinics of University	Isfahan, Iran	McDonald	Questionnaire	119/593	29.2 ± 9	20.1	43.5	32°39′N	NR	Middle East	3.76	6
Sahraian, 2010 [44]	2002–2008	Cross-sectional/ Iranian MS Society registry system	Tehran, Iran	McDonald and Poser	Questionnaire	773/8146	NR	9.5	51.9	32° 00′ N	NR	Middle East	NR	6
Kułakowska, 2010 [45]	2008–2009	Cross-sectional/ 21 centers providing MS treatment	Poland	McDonald	Questionnaire	184/2871	NR	6.4	57	52° 00′ N	NR	Europe	NR	6
Yamout, 2008 [46]	2005–2007	Cohort/ Lebanese registry of MS	Lebanon	McDonald	Medical records and questionnaire	10/202	NR	5	25	33°00′N	Nobody	Middle East	NR	5
Taraghi, 2007 [47]	2005–2006	Cross-sectional/ Mazandaran MS Association	Mazandaran, Iran	McDonald	Questionnaire	7/101	NR	7	27.9	36°30′N	NR	Middle East	NR	5
Saadatnia, 2007 [48]	2003–2006	Cross-sectional/ Isfahan MS Society registry system	Isfahan, Iran	McDonald	Medical records	209/1718	NR	12.2	43.8	32°39′N	NR	Middle East	2.7	7
Fricska-Nagy, 2007 [49]	2004	Cross-sectional/ five Hungarian MS centers	Hungary	McDonald	Medical records	33/1500	NR	2.2	62	47° 00′ N	NR	Europe	2.6	6
Peterlin, 2006 [50]	1999	Cross-sectional/ National registry and multi-center clinics	Gorski kotar– Kočevje, neighboring regions of the Republics of Croatia and Slovenia, respectively	Poser’s	Medical records	25/87	NR	28.7	151.9	45° 15′ N	NR	Europe	NR	7
Etemadifar, 2006 [51]	2004–2005	Cross-sectional/ Isfahan MS Society registry system	Isfahan, Iran	McDonald	Questionnaire and medical records	161/1391	NR	11.6	35.5	32°39′N	NR	Middle East	NR	7
Deryck, 2006 [52]	1987–2003	Cohort/ National MS Centre and Neurology department of the University	Belgium	Poser	Medical records	9/49	NR	18.4	68	50° 50′ N	All < 16	Europe	NR	6
Ebers, 2000 [53]	1972–1997	Cohort/ University Clinic	London, Ontario, Canada	Poser	Questionnaire and medical records	208/1044	NR	19.9	160	42°59′N	NR	North America	1.8	5
Gonzalez, 1995 [54]	1973–1992	Cross-sectional/ National Institute of Neurology and Neurosurgery	Mexico	Poser	Medical records	9/272	NR	3.3	1.6	19.00° N	NR	Central America	NR	6
Hader, 1988 [55]	1974–1983	Cross-sectional, Multi-center Hospital	London, Ontario, Canada	Schumacher	Medical records and Questionnaire	39/229	NR	17	88	42°59′N	NR	North America	NR	7
Wikstrom, 1984 [56]	1964–1979	Cross-sectional/ Both national registry and central hospitals	Jalasjarvi District of Vaasa, Finland	Schumacher	Questionnaire	15/51	33.7 ± 8.7	29.4	101	63.09° N	NR	Europe	NR	6

*yr* Year, *NR* not reported

^a^Repetitive studies have provided the data for both pediatric-onset and adult-onset MS

^b^In studies spanning decades, a mixed version of MacDonald criteria were used

^c^The information on latitude was gathered from this website: https://www.mapsofworld.com/

### Meta-analysis of whole data

**Fig. 2:**
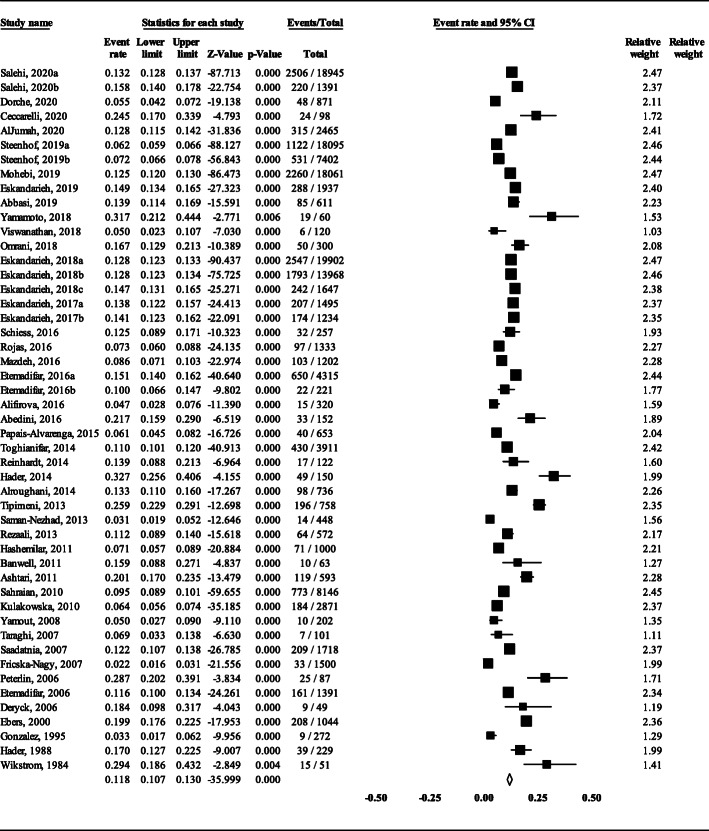
The prevalence of FMS (Heterogeneity: I^2^ = 97.112%, *P* < 0.001, Random effects)

**Fig. 3:**
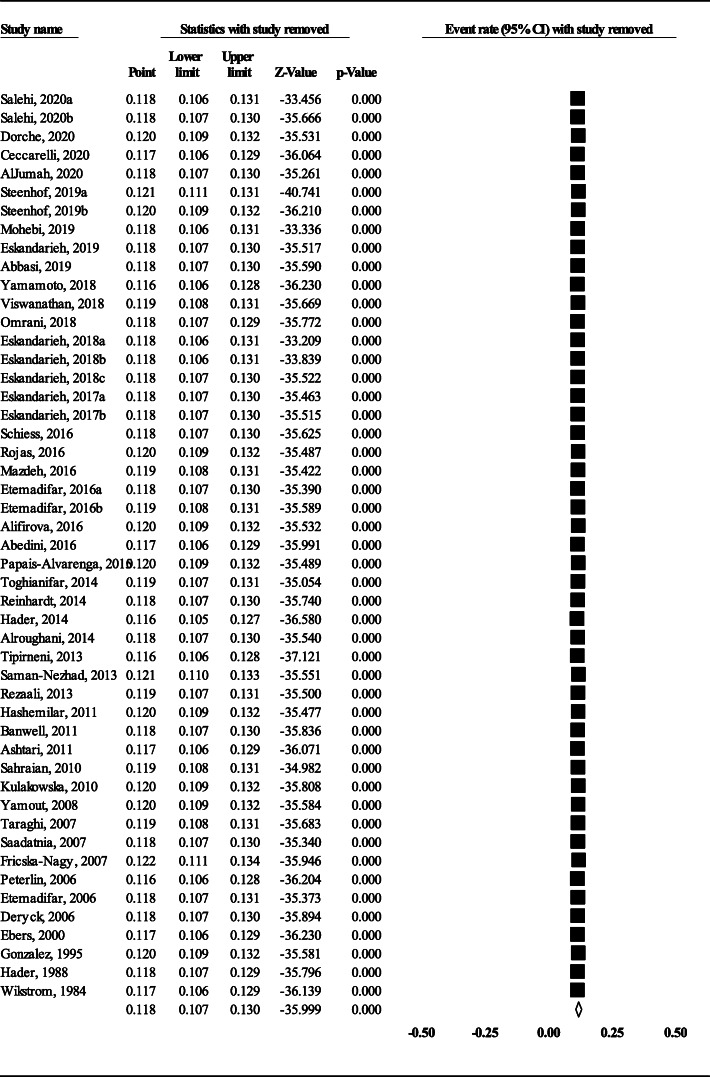
Forest plot of sensitivity analysis

Because of high total heterogeneity (Q = 1662.2, I^2^ = 97.112% and *P* < 0.001), a random-effects model was used. The polled prevalence of FMS was estimated to be 11.8% (95% CI: 10.7–13) of the total MS population (Fig. 2). The highest and lowest prevalence was found in Saskatchewan of Canada (32.7%) [36] and Hungary (2.2%) [49], respectively. The sensitivity analysis indicated our robust pooled estimate (Fig. 3).

### Meta-analysis of mean age of onset in AOMS and prevalence of FMS in AOMS and POMS

**Fig. 4:**
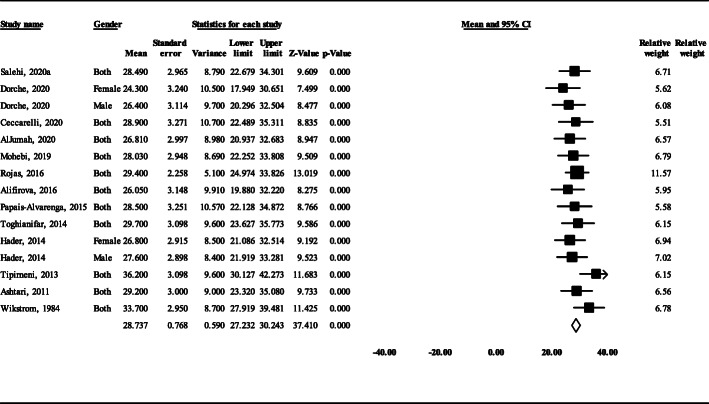
Mean age of disease onset in FMS cases

**Fig. 5:**
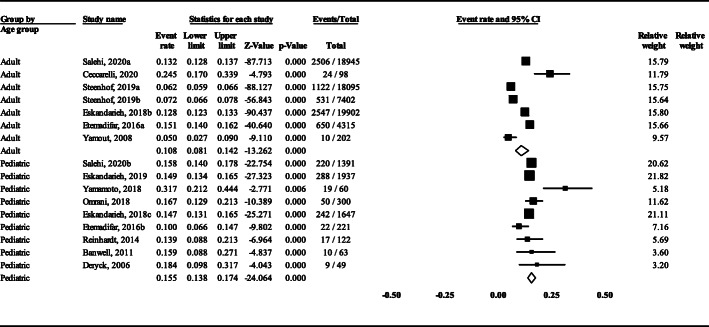
The prevalence of FMS in POMS and AOMS cases

The pooled mean age of disease onset in AOMS probands of 15 studies (*n* = 6114) that reported this variable was 28.7 years (95% CI: 27.2 ± 30.2) (Fig. 4). In this regard, the lowest and highest age of disease onset was recorded in Shiraz city of Iran (24.3 years) and New York of USA (36.2 years), respectively. In 13 studies that reported the data of AOMS (*n* = 6636) and POMS (*n* = 877), the FMS prevalence in AOMS was 10.8% (95% CI: 8.1–14.2) and in POMS was 15.5% (95% CI: 13.8–17.4), respectively (Fig. 5). The difference between these two groups was statistically significant (*P* = 0.019).

### Meta-analysis of FMS prevalence in men and women and OR of male/female

**Fig. 6:**
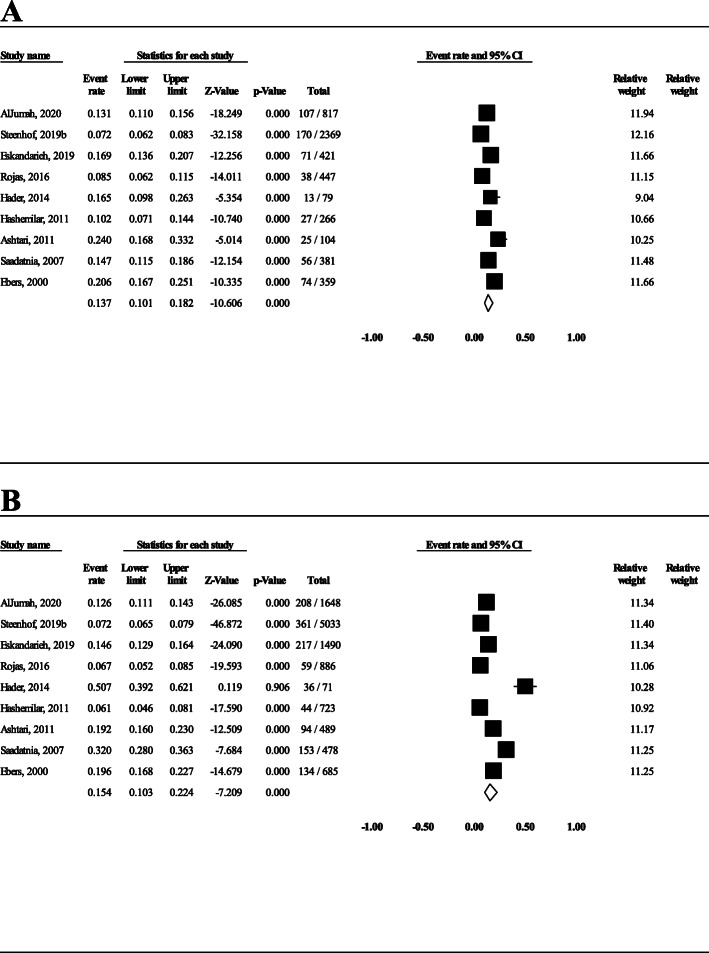
The prevalence of FMS in men (**A**) and women (**B**)

**Fig. 7:**
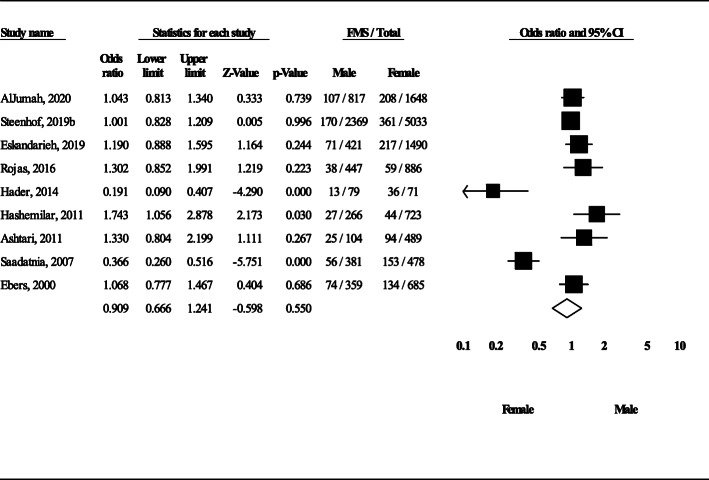
The OR of male/female among FMS cases

Nine studies provided the data of FMS and sporadic cases for men and women separately. Therefore, it was possible to calculate FMS prevalence for each sex separately. With regard to data of the 9 studies, the prevalence of FMS in affected males (*n* = 5243) and females (*n* = 11,503) was calculated to be 13.7% (95% CI: 10.1–18.2) and 15.4% (95% CI: 10.3–22.4), respectively (Fig. 6). In Fig. 6, in each study, the first number is the number of FMS cases and the second number is the number of FMS plus sporadic cases (both in that particular gender). For instance, in the study of AlJumah et al., 107 men and 208 women with FMS and 710 men and 1440 women with sporadic MS were recruited [16]; therefore, the rate of FMS in men and women was calculated to be 107/817 (710 + 107) and 208/1648 (208 + 1440), respectively. The OR of male/female in FMS cases was not statistically significant (OR = 0.9; 95% CI: 0.6–1.2, *P* = 0.55) (Fig. 7). In Additional file 3, detailed data of each 9 studies and how the odds ratio was calculated is presented.

### Subgroup analysis and meta-regression

**Fig. 8:**
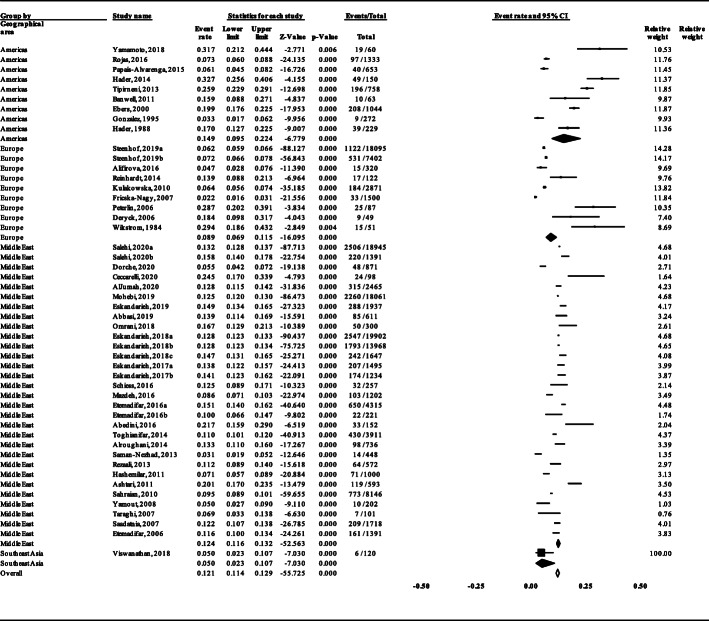
The prevalence of FMS in different geographical areas

Subgroup analysis revealed a significant difference in the prevalence of FMS between the geographical areas (Test for subgroup differences: Q = 12.070, df(Q) = 3, *P* = 0.007) (Fig. 8).

**Fig. 9:**
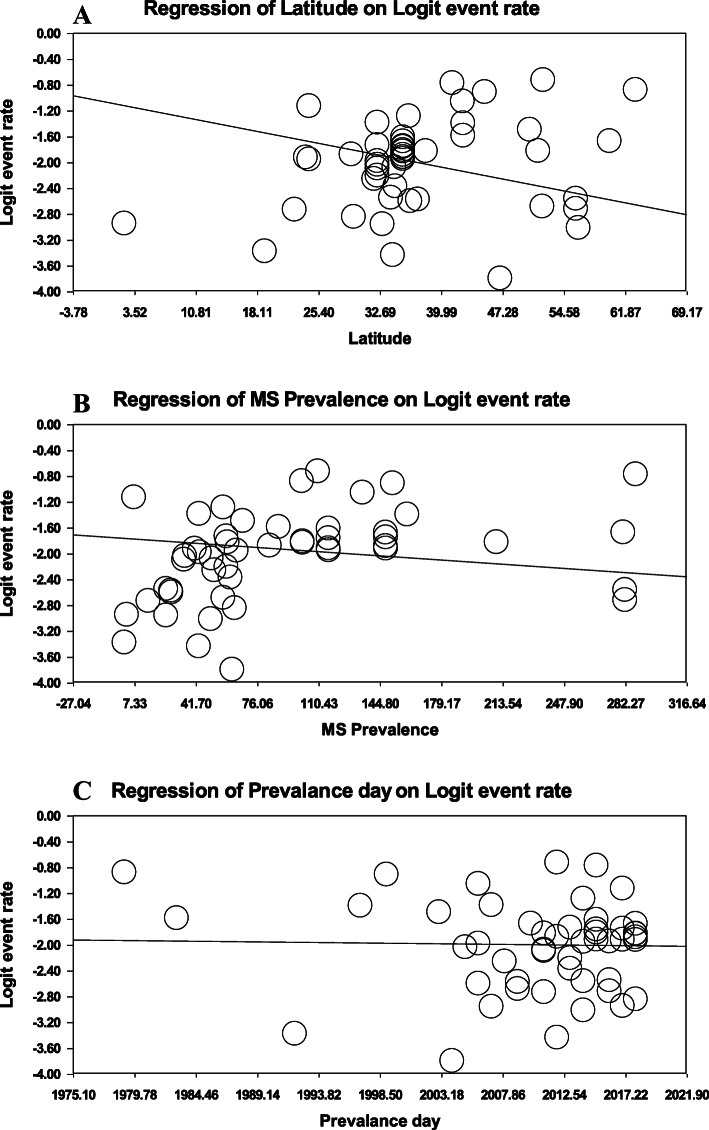
Meta-regression analysis of FMS prevalence in terms of latitude (meta-regression coefficient: -0.025, 95% CI: -0.027 to -0.023, *P*< 0.001) (**A**), MS prevalence (meta-regression coefficient: -0.0018, 95% CI: -0.0021 to -0.0016, *P*< 0.001) (**B**), and prevalence day (meta-regression coefficient: -0.002, 95% CI: -0.005 to 0.001, *P*=0.29) (**C**)

The meta-regression model showed that FMS prevalence was significantly lower in higher latitude (meta-regression coefficient: -0.025, 95% CI: − 0.027 to − 0.023, *P* < 0.001) (Fig. 9A). Similarly, a slight downward trend was observed with higher MS prevalence (meta-regression coefficient: -0.0018, 95% CI: − 0.0021 to − 0.0016, *P* < 0.001) (Fig. 9B). While, meta-regression based on prevalence day was not statistically significant (meta-regression coefficient: -0.002, 95% CI: − 0.005 to 0.001, *P* = 0.29) (Fig. 9C).

### Publication bias

**Fig. 10:**
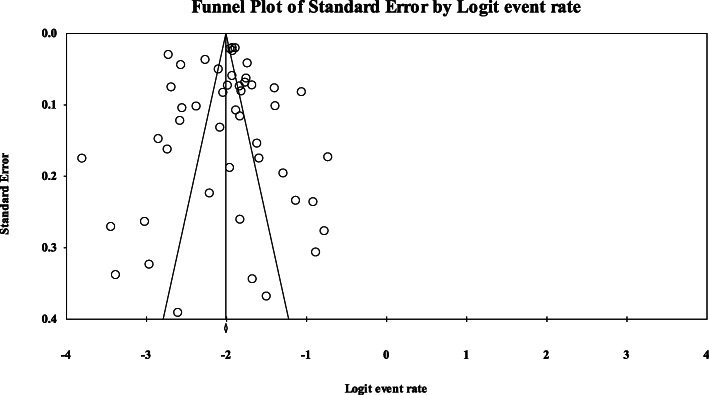
Funnel plot of publication bias

No publication bias was found in our analysis (Egger = 0.98, and Begg’s = 0.25) as depicted in the funnel plot (Fig. 10).

## Discussion

The results of a previous meta-analysis indicated that MS can run in families [57]. The pooled prevalence of FMS in our study (11.8%) (Fig. 2) was lower than the previous meta-analysis (12.6%) [9]. Hence, we performed a meta-regression analysis based on prevalence day to examine if the prevalence of the FMS has been decreased over time. Our results showed a non-significant lowering trend (Fig. 9C). Hence, it seems that the worldwide frequency of FMS is steady-state over time. Nonetheless, some studies in middle-east have reported the increasing [13, 24] or decreasing [16] prevalence of FMS over time. The overwhelming majority of the studies have been performed in a cross-sectional setting; while, a sufficiently long follow-up period is needed to evaluate the development of the disease in new members of the relatives. In this regard, cohort studies could be well suited on the account of a longer time period for the accumulation of new cases in the family.

POMS is defined as the manifestation of MS symptoms under the age of 16 or 18 [58]. According to our analysis, the frequency of FMS in POMS was higher than AOMS (Fig. 5). However, only 3 to 10% of sporadic cases have been reported to be POMS [59]. This informs us that increased genetic load may be a pivotal feature of POMS and the family history of MS could be a crucial contributing factor for POMS predisposition. Considering that association between HLA-DRB1*15:01 and age at onset shows a 10.6 months reduction in age at onset with each DRB1*15:01 allele [60], the difference between POMS and AOMS could be attributed to the higher frequency of DRB1*15:01 in POMS compared with AOMS [61]. By considering follow-up time bias, it seems that the prevalence of FMS is underestimated in the pediatric group due to not emergence of this disease in relatives especially siblings at the time of the study, at least in cross-sectional studies.

The mean age of onset in adult probands with FMS was estimated to be 28.7 (Fig. 4), which indicates an earlier age of onset among FMS cases in comparison to sporadic cases [7, 62]. This highlights the point that the preclinical phase of the disease would be shortened in cases with higher genetic load and consequently, symptoms initiate at a lower age at onset. In this regard, as previously mentioned, several studies have indicated that HLA, especially HLA-DRB1*15:01, is the main genetic influence on age of disease onset [63–65].

Given the concept of the “carter effect” [66], we set out to investigate the notion that in male MS patients, the prevalence of FMS is more than in females patients, as well as transmission to other members of the family, is higher when the affected individual is male. However, the prevalence of FMS in male and female cases and OR of male/female FMS cases did not confirm this theory (Figs. 6 and 7). This represents that a greater than average background of susceptibility factors in an affected male which is the less frequently affected sex does not increase the occurrence of the MS in relatives. On the contrary, a higher prevalence of FMS and positive family in males than that in females was seen in the Iranian population [67]. Regarding this discrepancy, we acknowledge that the low sample size for scrutinizing the effect of sex may lead to this interpretation.

Subgroup analysis unveiled that the distribution of FMS is different between geographical areas (Fig. 8). However, even between different studies in the same geographical area, the FMS prevalence is different, mirroring that this difference is more complicated than can be explained only by just differences in susceptibility between racial and ethnic groups. Also, this finding could justify the high heterogeneity between studies, at least in part. Relevantly, other meta-analysis indicated different FMS prevalence in Iran (8.9%) [67] and the Middle East North Africa region (17.8%) [68].

It is expected that with the increasing prevalence of sporadic MS, the frequency of FMS rises, as well. Quite interestingly, our meta-regression analysis revealed a weak decreasing trend of FMS with higher MS prevalence (Fig. 9A). In the same vein, meta-regression in terms of latitude disclosed that the prevalence of FMS is decreased in conjunction with an increment of latitude (Fig. 9B); although, traditionally, MS has been more prevalent in regions at higher latitudes with decreased sunlight exposure [69]. Intriguingly, without conducting a statistical analysis, Harirchian and coworkers concluded that the prevalence of FMS was not latitude dependent. We addressed this issue in our study and found that at odds with the previous study the prevalence of FMS is latitude dependent. Thereby, we hypothesized that with the increasing frequency of MS in a region, the public awareness and familiarity of the people, especially genetic counselors, with the disease grows, too. Therefore, the rate of marriages in which one or both sides have one or more affected members reduces. This, in turn, lowers the shared genetic variants in families. On the other hand, the rate of consanguineous marriage as a predictor of positive family history of MS [16], will most probably be diminished in regions with a high outbreak of this disease.

In comparison to the previous systematic review [9], the strengths of our study were recruiting of a quality assessment tool for inclusion of studies, no limitation of language for searching of articles, uncovering the prevalence of FMS in different geographical areas, in POMS and AOMS cases, and men and women, unveiling the relationship between the prevalence of FMS and prevalence day, MS prevalence and latitude, determining the mean age of the disease onset in adult probands and the effect of gender on FMS occurrence. However, we acknowledge that it would have been better if other databases such as Embase had been examined. Notwithstanding, there are some issues in the included studies which mostly are derived from the retrospective design. For instance, recall bias could occur when the presence of affected relatives is assessed by employing questionnaires and medical records which hinges on patients’ self-reporting. This might result in the under-diagnosis of distant relatives. On the other hand, the diversity in case ascertainment methodology namely population (registry or community)-based or clinical (hospital)-based may cause the sampling bias.

## Conclusion

In summary, the findings of this study demonstrated that the prevalence of FMS is higher in POMS cases than that of AOMS, is different between geographical areas, and reduces with the higher MS prevalence and latitude. Likewise, the symptoms embark relatively at lower ages in FMS probands of AOMS. Unexpectedly, the prevalence of FMS was not more prevalent in men than women and the risk of MS development in relatives was not higher when the affected proband was male. For preventing biases, we suggest that future studies be performed as longitudinal prospective to provide time for the development of new cases in relatives. Also, the reported affected members of the family must be reexamined by neurologists.

## Supplementary Information

**Additional file 1.** The search strategy that was used in the databases.

**Additional file 2.** Excluded studies with reason.

**Additional file 3.** The data of 9 studies that provided the number of familial and sporadic cases for men and women to calculate the prevalence of FMS separately for each gender and also the odds ratio (Section 3.4).

## Data Availability

All data generated or analysed during this study are included in this published article [and its supplementary information files].

## Abbreviations

MS: Multiple sclerosis
FMS: Familial multiple sclerosis
POMS: Pediatric-onset MS
AOMS: Adult-onset MS
PRISMA: Preferred Reporting Items for Systematic Reviews and Meta-Analyses
CoCoPop: Condition, context, and population
OR: Odds ratio
