# Supplementary material for: The global prevalence of familial multiple sclerosis: an updated systematic review and meta-analysis
Source: BMC Neurol. 2021 Jun 28;21:246. doi: 10.1186/s12883-021-02267-9 (PMC8237453; doi:10.1186/s12883-021-02267-9)
Supplement: Supplementary file 1 — Additional file 1. The search strategy that was used in the databases. [file 12883_2021_2267_MOESM1_ESM.docx]

**The global prevalence of familial multiple sclerosis: an updated systematic review and meta-analysis**

Naeim Ehtesham ^1&2*^, Maryam Zare Rafie ^3^, Meysam Mosallaei ^2^

1. Student Research Committee, University of Social Welfare and Rehabilitation Sciences , Tehran , Iran
2. Genetics and Molecular Biology, School of Medicine, Isfahan University of Medical Sciences, Isfahan, Iran
3. Zanjan University of Medical Sciences, Zanjan, Iran

***Corresponding author:** Naeim Ehtesham

**Affiliations:**

- Student Research Committee, University of Social Welfare and Rehabilitation Sciences, Tehran, Iran
- Department of Genetics and Molecular Biology, School of Medicine, Isfahan University of Medical Sciences, Isfahan, Iran

**Email:** na.ehtesham@uswr.ac.ir; Naeim.ehtesham@yahoo.com

**ORCiD:** 0000-0002-1769-6329

**Tel:** (+98)-21 7173 2833

**Fax:** (+98)-21 7173 4516

**Postal address**: Koodakyar Alley, Daneshjoo Blvd., Evin St., Tehran, Iran

**Additional file 1:** The search strategy that was used in the databases (Date: 20/12/2020):

**PubMed:**

("Multiple sclerosis"[Title/Abstract]) AND ("Familial"[Text Word]) AND ("epidemiology"[Title/Abstract] OR "prevalence"[Title/Abstract] OR "incidence"[Title/Abstract] OR "recurrence"[Title/Abstract] OR "frequency"[Title/Abstract])

Number: 178

**Scopus:**

( TITLE-ABS-KEY ( "Multiple sclerosis" ) )  AND  ( TITLE-ABS-KEY ( "Familial" ) )  AND  ( TITLE-ABS-KEY ( "epidemiology" )  OR  TITLE-ABS-KEY ( "prevalence" )  OR  TITLE-ABS-KEY ( "incidence" )  OR  TITLE-ABS-KEY ( "recurrence" )  OR  TITLE-ABS-KEY ( "frequency" ) )

Number: 320

**ISI Web of Science:**

#1: TOPIC:  ("Multiple sclerosis"), # 2: TOPIC:  ("Familial"), #3: TOPIC:  ("epidemiology")  OR  TOPIC:  ("prevalence")  OR  TOPIC:  ("incidence")  OR  TOPIC:  ("recurrence")  OR  TOPIC:  ("frequency"), #4: #3 AND #2 AND #1

Number: 241
