# Supplementary material for: The global prevalence of familial multiple sclerosis: an updated systematic review and meta-analysis
Source: BMC Neurol. 2021 Jun 28;21:246. doi: 10.1186/s12883-021-02267-9 (PMC8237453; doi:10.1186/s12883-021-02267-9)
Supplement: Supplementary file 2 — Additional file 2. Excluded studies with reason. [file 12883_2021_2267_MOESM2_ESM.docx]

**The global prevalence of familial multiple sclerosis: an updated systematic review and meta-analysis**

Naeim Ehtesham ^1&2*^, Maryam Zare Rafie ^3^, Meysam Mosallaei ^2^

1. Student Research Committee, University of Social Welfare and Rehabilitation Sciences , Tehran , Iran
2. Genetics and Molecular Biology, School of Medicine, Isfahan University of Medical Sciences, Isfahan, Iran
3. Zanjan University of Medical Sciences, Zanjan, Iran

***Corresponding author:** Naeim Ehtesham

**Affiliations:**

- Student Research Committee, University of Social Welfare and Rehabilitation Sciences, Tehran, Iran
- Department of Genetics and Molecular Biology, School of Medicine, Isfahan University of Medical Sciences, Isfahan, Iran

**Email:** na.ehtesham@uswr.ac.ir; Naeim.ehtesham@yahoo.com

**ORCiD:** 0000-0002-1769-6329

**Tel:** (+98)-21 7173 2833

**Fax:** (+98)-21 7173 4516

**Postal address**: Koodakyar Alley, Daneshjoo Blvd., Evin St., Tehran, Iran

**Additional file 2:** Excluded studies with reason

| **Reason** | **First author, Published Year** | **Title** |
| --- | --- | --- |
| Duplicate | Eskandarieh, 2020 | Risk Factors and Prevalence of Familial Multiple Sclerosis: A Population- Registry Based Study |
|  | Almasi-Hashiani, 2020 | Evidence of an increased prevalence of multiple sclerosis: a population-based study of Tehran registry during 1999-2018 |
|  | Titova, 2016 | Epidemiology of familial cases of multiple sclerosis in Tomsk region, Russian Federation |
|  | Eskandarieh, 2016 | Increasing prevalence and decreasing disease onset among of familial multiple sclerosis in Tehran, Iran |
|  | Taraghi, 2010 | Quality of Life among Multiple Sclerosis Patients |
|  | Kinnunen, 1984 | Prevalence, incidence and familial risk of multiple-sclerosis |
| Consideration of more than one region | Yamout, 2020 | Epidemiology and phenotypes of multiple sclerosis in the Middle East North Africa (MENA) region |
|  | Nasehi, 2017 | Clinical and Epidemiological Aspects of Multiple Sclerosis in Children |
|  | Renoux, 2007 | Natural history of multiple sclerosis with childhood onset |
| Not determination of prevalence day | Al Jumah, 2011 | Familial multiple sclerosis: does consanguinity have a role? |
|  | Fernández-Pérez, 1999 | Clinical features of familial multiple sclerosis in Spain |
|  | Duquette, 1987 | Multiple sclerosis in childhood: clinical profile in 125 patients |
|  | Ebers, 1983 | Genetic factors in multiple sclerosis |
|  | Roberts, 1982 | The genetic contribution to multiple sclerosis. Evidence from North-East England |
|  | Sadovnick, 1981 | The familial nature of multiple sclerosis: empiric recurrence risks for first, second-, and third-degree relatives of patients |
| Inclusion of NMO or ADS in addition to MS | Bonnan, 2020 | Familial clustering of neuromyelitis optica and multiple sclerosis: clues pointing towards shared risks? |
|  | Inaloo, 2014 | Acquired CNS Demyelinating Syndrome in Children Referred to Shiraz Pediatric Neurology Ward |
|  | Moghtaderi, 2013 | Incidence and prevalence of multiple sclerosis in southeastern Iran |
|  | Banwell, 2009 | Incidence of acquired demyelination of the CNS in Canadian children |
|  | Kuroiwa, 1975 | Nationwide survey of multiple sclerosis in Japan. Clinical analysis of 1,084 cases |
| Regard of two or more populations and time periods | Pratt, 1951 | The familial incidence of disseminated sclerosis and its significance |
|  | Pratt, 1951 | The familial occurrence of disseminated sclerosis |
| Not available of the full-text | Sazdovitch, 2000 | Familial multiple sclerosis: study of 357 consecutive patients |
| Inclusion of probable and possible cases | O'Gorman, 2011 | Familial recurrence risks for multiple sclerosis in Australia |
|  | Romero-Pinel, 2010 | Anticipation of age at onset in familial multiple sclerosis |
|  | Pohl, 2007 | Paediatric multiple sclerosis and acute disseminated encephalomyelitis in Germany: results of a nationwide survey |
|  | Hader, 2007 | Incidence and prevalence of multiple sclerosis in Saskatoon, Saskatchewan |
|  | Etemadifar, 2007 | Childhood-onset multiple sclerosis: report of 82 patients from Isfahan, Iran |
|  | El-salem, 2007 | Comparison of the natural history and prognostic features of early onset and adult onset multiple sclerosis in Jordanian population |
|  | El-Salem, 2006 | Multiple sclerosis in Jordan: A clinical and epidemiological study |
|  | Al-araji, 2005 | Multiple sclerosis in Iraq: does it have the same features encountered in Western countries? |
|  | Ozakbas, 2003 | Childhood and juvenile onset multiple sclerosis: clinical and paraclinical features |
|  | Daif, 1998 | Pattern of presentation of multiple sclerosis in Saudi Arabia: analysis based on clinical and paraclinical features |
|  | Carton, 1997 | Risks of multiple sclerosis in relatives of patients in Flanders, Belgium |
|  | Shepherd, 1996 | Prevalence of multiple sclerosis in Rochdale |
|  | Robertson, 1996 | Age-adjusted recurrence risks for relatives of patients with multiple sclerosis |
|  | Sharpe, 1995 | Multiple sclerosis in island populations: prevalence in the Bailiwicks of Guernsey and Jersey |
|  | Al-Din, 1995 | Multiple sclerosis in Arabs in Jordan |
|  | Alvarez, 1992 | Multiple sclerosis in Chile |
|  | Weinshenker, 1990 | A comparison of sporadic and familial multiple sclerosis |
|  | Sadovnick, 1988 | Multiple sclerosis: updated risks for relatives |
|  | Phadke, 1987 | Epidemiology of multiple sclerosis in the north-east (Grampian Region) of Scotland - an update |
|  | Poser, 1979 | Clinical data and the identification of special forms of multiple sclerosis in 1271 cases studied with a standardized documentation system |
|  | Morariu, 1974 | Multiple sclerosis in transylvania: A zone of transition in frequency |
|  | Gudmundsson, 1974 | Further studies on multiple sclerosis in iceland |
|  | Schapira, 1963 | Familial and conjugal multiple sclerosis |
|  | Sutherland, 1956 | Observations on the prevalence of multiple sclerosis in Northern Scotland |
|  | Millar, 1954 | Familial incidence of disseminated sclerosis in Northern Ireland |
| Not determination of target population | Viswanathan, 2013 | Multiple Sclerosis in Malaysia: Demographics, Clinical Features, and Neuroimaging Characteristics |
| Low quality | Chinea, 2018 | Family distribution in multiple sclerosis: a study of the prevalence of familial recurrence in Puerto Rico |
|  | Khoshravesh, 2016 | Awareness, risk perception, and protective behaviors in regard to multiple sclerosis among people in Sanandaj, Iran |
|  | Esposito, 2015 | Impact of MS genetic loci on familial aggregation, clinical phenotype, and disease prediction |
|  | Farez, 2014 | Low familial risks for multiple sclerosis in Buenos Aires, Argentina |
|  | Deleu, 2013 | Prevalence, demographics and clinical characteristics of multiple sclerosis in Qatar |
|  | Danesh-Sani, 2013 | Clinical assessment of orofacial manifestations in 500 patients with multiple sclerosis |
|  | Baghizadeh, 2013 | Clinical and demographic factors affecting disease severity in patients with multiple sclerosis |
|  | Pourmemari, 2011 | Epidemiologic variables in Multiple sclerosis patients in Zanjan |
|  | Inshasi, 2011 | Prevalence of multiple sclerosis in Dubai, United Arab Emirates |
|  | Sawaya, 2009 | Multiple sclerosis in Lebanon: a review of 45 cases |
|  | Mazaheri, 2008 | Clinical and epidemiological features of early and adult onset multiple sclerosis in Hamedan, Iran, 2004 2005 |
|  | Koch, 2008 | Progression in familial and nonfamilial MS |
|  | Siritho, 2007 | A retrospective study of multiple sclerosis in Siriraj Hospital, Bankok, Thailand |
|  | Ghabaae, 2007 | Epidemiology of multiple sclerosis in Tehran: a three year study |
|  | Kouroumalos, 2005 | Familial incidence of multiple sclerosis in Chania, Crete |
|  | Amela-Peris, 2004 | Familial multiple sclerosis in Canary Islands |
|  | Zorzon, 2003 | Risk factors of multiple sclerosis: a case-control study |
|  | Kalanie, 2003 | Multiple sclerosis: report on 200 cases from Iran |
|  | Simone, 2002 | Course and prognosis in early-onset MS: comparison with adult-onset forms |
|  | Yu, 1989 | Multiple sclerosis amongst Chinese in Hong Kong |
|  | Tan, 1988 | Multiple Sclerosis in Malaysia |
|  | Shibasaki, 1981 | Racial modification of clinical picture of multiple sclerosis: comparison between British and Japanese patients |
|  | Wikstrom, 1975 | Studies on the clustering of multiple sclerosis in Finland II: microepidemiology in one high-risk county with special reference to familial cases |
|  | Cendrowski, 1967 | Familial multiple sclerosis in Poland. Part 1. Geographical distribution of the disease |

NMO: neuromyelitis optica; ADS: Acquired demyelinating syndromes
