# Supplementary material for: The global prevalence of familial multiple sclerosis: an updated systematic review and meta-analysis
Source: BMC Neurol. 2021 Jun 28;21:246. doi: 10.1186/s12883-021-02267-9 (PMC8237453; doi:10.1186/s12883-021-02267-9)
Supplement: Supplementary file 3 — Additional file 3. The data of 9 studies that provided the number of familial and sporadic cases for men and women to calculate the prevalence of FMS separately for each gender and also the odds ratio (Section 3.4). [file 12883_2021_2267_MOESM3_ESM.docx]

**The global prevalence of familial multiple sclerosis: an updated systematic review and meta-analysis**

Naeim Ehtesham ^1&2*^, Maryam Zare Rafie ^3^, Meysam Mosallaei ^2^

1. Student Research Committee, University of Social Welfare and Rehabilitation Sciences , Tehran , Iran
2. Genetics and Molecular Biology, School of Medicine, Isfahan University of Medical Sciences, Isfahan, Iran
3. Zanjan University of Medical Sciences, Zanjan, Iran

***Corresponding author:** Naeim Ehtesham

**Affiliations:**

- Student Research Committee, University of Social Welfare and Rehabilitation Sciences, Tehran, Iran
- Department of Genetics and Molecular Biology, School of Medicine, Isfahan University of Medical Sciences, Isfahan, Iran

**Email:** na.ehtesham@uswr.ac.ir; Naeim.ehtesham@yahoo.com

**ORCiD:** 0000-0002-1769-6329

**Tel:** (+98)-21 7173 2833

**Fax:** (+98)-21 7173 4516

**Postal address**: Koodakyar Alley, Daneshjoo Blvd., Evin St., Tehran, Iran

**Additional file 3:** The data of 9 studies that provided the number of familial and sporadic cases for men and women to calculate the prevalence of FMS separately for each gender and also the odds ratio (Section 3.4 of the article).

| **First author, Published Year** | **Men** | | | | | **Women** | | | | |
| --- | --- | --- | --- | --- | --- | --- | --- | --- | --- | --- |
|  | **Familial** | **Sporadic** | | | **Total** | **Familial** | **Sporadic** | | | **Total** |
| AlJumah, 2020 [1] | 107 | | 710 | 817 | | 208 | | 1440 | 1648 | |
| Steenhof, 2019b [2] | 170 | | 2199 | 2369 | | 361 | | 4672 | 5033 | |
| Eskandarieh, 2019 [3] | 71 | | 350 | 421 | | 217 | | 1273 | 1490 | |
| Rojas, 2016 [4] | 38 | | 409 | 447 | | 59 | | 827 | 886 | |
| Hader, 2014 [5] | 13 | | 66 | 79 | | 36 | | 35 | 71 | |
| Hashemilar, 2011 [6] | 27 | | 239 | 266 | | 44 | | 679 | 723 | |
| Ashtari, 2011 [7] | 25 | | 79 | 104 | | 94 | | 395 | 489 | |
| Saadatnia, 2007 [8] | 56 | | 325 | 381 | | 153 | | 325 | 478 | |
| Ebers, 2000 [9] | 74 | | 285 | 359 | | 134 | | 551 | 685 | |

The odds ratio for each study was calculated by comprehensive meta-analysis according to this formula (The data of AlJumah, 2020 was used as an example):

A = FMS Male

B = FMS Female

C = Sporadic Male

D = Sporadic Female

LogOddsRatio = Log((A * D) / (B * C))

LogOddsVariance = (1 / A + 1 / B + 1 / C + 1 / D)

LogOddsSE = Sqr(LogOddsVariance)

OddsRatio = Exp(LogOddsRatio)

LogOddsRatio = Log((107 * 1440) / (208 * 710)) = 0.042

LogOddsVariance = (1/107 + 1/208 + 1/710 + 1/1440) = 0.016

LogOddsSE = Sqr(0.016) = 0.128

OddsRatio = Exp(0.042) = 1.043

**References**

1. AlJumah M, Otaibi HA, Al Towaijri G, Hassan A, Kareem A, Kalakatawi M et al. Familial aggregation of multiple sclerosis: Results from the national registry of the disease in Saudi Arabia. Mult Scler J Exp Transl Clin. 2020;6(4):2055217320960499. doi:10.1177/2055217320960499.

2. Steenhof M, Nielsen NM, Stenager E, Kyvik K, Möller S, Hertz JM. Distribution of disease courses in familial vs sporadic multiple sclerosis. Acta Neurol Scand. 2019;139(3):231-7. doi:10.1111/ane.13044.

3. Eskandarieh S, Sahraiain MA, Molazadeh N, Moghadasi AN. Pediatric multiple sclerosis and its familial recurrence: A population based study (1999–2017). Multiple Sclerosis and Related Disorders. 2019;36. doi:10.1016/j.msard.2019.101377.

4. Rojas JI, Patrucco L, J MI, Sinay V, Cassara FP, Cáceres F et al. Disease onset in familial and sporadic multiple sclerosis in Argentina. Mult Scler Relat Disord. 2016;6:54-6. doi:10.1016/j.msard.2016.01.004.

5. Hader WJ, Yee IM. The prevalence of familial multiple sclerosis in saskatoon, Saskatchewan. Mult Scler Int. 2014;2014:545080. doi:10.1155/2014/545080.

6. Hashemilar M, Ouskui DS, Farhoudi M, Ayromlou, Asadollahi A, editors. Multiple sclerosis in East Azerbaijan, North West Iran2011.

7. Ashtari F, Shaygannejad V, Heidari F, Akbari M. Prevalence of familial multiple sclerosis in Isfahan, Iran. Journal of Isfahan Medical School. 2011;29(138):555-61.

8. Saadatnia M, Etemadifar M, Maghzi AH. Multiple sclerosis in Isfahan, Iran. Int Rev Neurobiol. 2007;79:357-75. doi:10.1016/s0074-7742(07)79016-5.

9. Ebers GC, Koopman WJ, Hader W, Sadovnick AD, Kremenchutzky M, Mandalfino P et al. The natural history of multiple sclerosis: A geographically based study. 8. Familial multiple sclerosis. Brain. 2000;123(3):641-9. doi:10.1093/brain/123.3.641.
